# Cerium‐Modified TiO_2_ Nanomaterials Derived From Upcycled Coffee Ground Waste for Photocatalytic Oxidation and Hydrogen Production via Photoreforming

**DOI:** 10.1002/open.70293

**Published:** 2026-09-21

**Authors:** Narimene Aoun, Elisa I. García‐López, Bartolomeo Megna, Chiara Aliotta, Valeria La Parola, Ganna Kovtun, Teresa Cuberes, Giuseppe Marcì

**Affiliations:** ^1^ Department of Engineering University of Palermo Palermo Italy; ^2^ Istituto per lo Studio dei Materiali Nanostrutturati (ISMN)‐CNR Palermo Italy; ^3^ Group of Nanotechnology and Materials Mining and Industrial Engineering School of Almadén University of Castilla‐La Mancha Almadén Spain; ^4^ V.G. Baryakhtar Institute of Magnetism of the NAS of Ukraine Kyiv Ukraine

**Keywords:** Ce–TiO_2_, coffee waste, photocatalysis, photoreforming, waste valorization

## Abstract

The valorization of spent coffee ground wastes to prepare advanced materials offers a promising pathway toward sustainable materials engineering. In this work, spent coffee grounds were valorized by their utilization as a template for the synthesis of Ce‐loaded TiO_2_ to be used as photocatalysts. The synthesis in the presence of the bio‐derived sacrificial template in the absence of solvent promote a hierarchical porosity and surface area and an intimate contact between Ce and Ti precursors to improve the photocatalytic activity of the obtained composite material. Pure TiO_2_ is made up of nanoparticles that organize into fairly uniform spherical agglomerates of ca. 1 μm. When Ce is introduced, this ordered structure gradually changes giving rise to less regular larger aggregates, and at higher loadings (5% Ce) the spherical morphology is no longer observed. In all cases a mesoporous structure is present. The activity of the obtained solids as photocatalysts was tested in three different reactions: (i) the photo reforming of ethanol in aqueous suspension of the photocatalysts in the absence of oxygen, to obtain hydrogen; (ii) the 2‐propanol oxidation in gas–solid regime, and (iii) the 5‑hydroxymethylfurfural (5‑HMF) oxidation in aqueous suspension of the solids.

## Graphical abstract

Spent coffee grounds were valorized as a sustainable template for the solvent‐free synthesis of Ce‐loaded TiO_2_ photocatalysts, promoting enhanced Ce–Ti interactions and tailored morphology. The resulting materials were successfully applied in photocatalytic hydrogen production via ethanol photoreforming, gas‐phase 2‐propanol oxidation, and aqueous 5‐hydroxymethylfurfural oxidation.

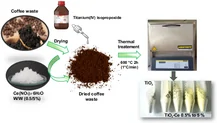

## 1 Introduction

The increasing generation of solid waste has increased the scientific interest in waste valorization within a circular economy framework. The coffee industry produces large amounts of residues such as spent coffee grounds, pulp, husk, and silver skin, which are rich in lignocellulosic and bioactive components and can be transformed into high‐value products. Biomass and organic waste are widely recognized as sustainable resources for biochar production [1]. In this context, coffee‐processing by‐products represent an abundant, low‐cost feedstock for bio‐based materials. Spent coffee grounds have been extensively used to produce porous biochars and heteroatom‐doped carbons. These materials exhibit promising catalytic performances and can act as metal‐free catalysts or as supports [2]. The sacrificial templating method is a widely used strategy for controlling materials morphology and porosity, thereby enhancing catalytic performance [3]. This materials synthesis strategy involves depositing a metal precursor onto a template, most commonly a surfactant, followed by template removal, typically through thermal treatment, to produce porous structures; notably, bio‐based wastes can also be employed as templates in this type of materials synthesis. Biomass‐derived templates such as wood, wheat bran, orange peels, and walnut shells have been successfully employed for the synthesis of nanostructured solids, yielding materials with promising catalytic properties [4].

The present study aims to exploit materials prepared by using biomass waste to obtain photocatalysts. In photocatalysis a chemical transformation occurs by virtue of the presence of a semiconductor, which is activated by light. This sustainable technology can be applied for water purification, CO_2_ reduction, H_2_ production, and green chemical synthesis at room conditions [5].

Some reports are devoted to the use of spent coffee wastes to obtain photocatalysts, for example, carbon nanoparticles for the photocatalytic degradation of tetracycline [6] or biochar for photocatalytic H_2_O_2_ generation [7]. Here, we have used the biomass waste as a templating agent to synthesize titania and Ce‐loaded titania composites, which are semiconductors and can be used as photocatalytic heterogeneous materials. Among potential applications of this technology we have investigated, the oxidation of organics in gas–solid regime; the oxidation of alcohols to the complete mineralization and/or the selective partial oxidation to obtain the correspondent aldehydes and ketones and eventually the H_2_ generation in liquid–solid regime by photoreforming of organics. All these reactions are of particular interest due to their relevance in the production of high‐value chemical compounds.

## 2 Experimental Section

### 2.1 Preparation and Characterization of the Photocatalysts

**FIGURE 1:**
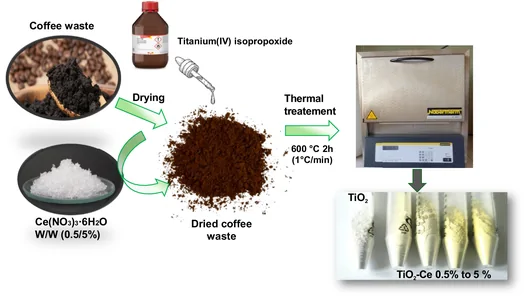
Schematic illustration of the synthesis process for Ce‐TiO_2_ nanocomposites.

The spent coffee grounds were washed with deionized water to eliminate soluble species, filtered, and dried in an oven at 80 °C for 24 h. The dried coffee waste was then ground and sieved to obtain a homogeneous particle size (ca. <500 µm). An amount of 10 g of dried sieved coffee waste was placed in glass beaker and 13 mL of pure titanium (IV) isopropoxide (TTIP) (Merck purity > 97%) was added dropwise under continuous stirring to ensure uniform wetting and impregnation of the biomass. The mixture was stirred for 30 min more to promote intimate contact between TTIP, and the coffee ground, forming a Ti‐loaded organic precursor. The mixture was then placed in an alumina crucible and introduced into a muffle furnace. The mixture was calcined during 2 h in static air at 600 °C (heating rate of 1 °C min^−1^). After calcination, the solid was carefully ground in an agate mortar into a fine powder. This material, derived from coffee waste and TTIP has been labeled TiO_2_ CW. Moreover, a series of Ce‐loaded TiO_2_ CW samples were prepared following the same described preparation steps, but the dried coffee ground waste (10 g) was carefully mixed up in a mortar with a measured amount of solid Ce(NO_3_)_3_⋅6H_2_O (Merck purity > 99.99%) before the addition of the 13 mL of TTIP and the samples obtained were labeled as TiO_2_ CW‐Ce 0.5%, 1%, 3%, and 5%, respectively, where the number indicates the nominal atomic percentage of Ce with respect to TiO_2_ moles in the samples obtained. The preparation process is schematized in Figure 1. To study the fate of CW during calcination, 5 g of CW were thermal treated like the other samples for 2 h at 600 °C (heating rate of 1 °C min^−1^) obtaining 0.06 g of ash and this powder was named ASH CW.

A detailed physicochemical characterization was carried out to better understand the properties of the photocatalysts. Their morphology, together with elemental composition, was examined by Scanning electron microscopy (SEM) coupled with EDX using a FEI Quanta 200 ESEM operating at 20 kV. Before analysis, the samples were coated with a thin gold layer in order to improve electrical conductivity. Moreover, the elemental composition of the coffee waste was analyzed by X‐ray fluorescence (XRF) using a Malvern Panalytical Epsilon 1 spectrometer based on energy dispersive XRF technology, with a spectral resolution of 135 eV (Mn Kα). The analysis was performed to determine the inorganic elemental composition of the biomass precursor. Transmission electron microscopy (TEM) and high‐resolution TEM (HRTEM) observations were performed using a JEOL JEM‐2100 transmission electron microscope (JEOL Ltd., Tokyo, Japan), equipped with a LaB_6_ thermionic electron source and operated at an accelerating voltage of 200 kV. For TEM analysis, the powder samples were dispersed in ethanol, and a drop of each suspension was deposited onto a lacey carbon‐coated TEM grid and allowed to dry before observation. Image analysis, lattice‐spacing measurements, and local fast Fourier transforms were performed using Gatan DigitalMicrograph software (version 3.7.4). To investigate the textural properties, nitrogen adsorption–desorption measurements were performed at −196 °C with an ASAP 2020 analyzer (Micromeritics, Norcross, GA, USA). The powders were first degassed at 250 °C for 2 h to remove any physisorbed species. From these measurements, the specific surface area was calculated using the BET method in the 0.05–0.30 (*P*/*P*_0_) range. Pore size distribution and total pore volume were instead obtained from the desorption branch, applying the BJH model [8]. Additional insight into the structure of the materials was obtained through Fourier transform infrared spectroscopy (FTIR) spectroscopy. The analyses were carried out on KBr pellets using a Shimadzu FTIR‐8400 instrument, with a resolution of 4 cm^−1^ and 256 accumulated scans.

X‐ray diffraction (XRD) patterns were collected using a Rigaku MiniFlex 600 powder diffractometer (Rigaku Europe SE, Neu‐Isenburg, Germany) equipped with Cu Kα radiation (40 kV, 15 mA) and a Ni filter. Data were acquired over the 2*θ* range of 20°–80° with a step size of 0.05° and a scan rate of 1° min^−1^. The qualitative structural analysis was performed with Database PDF‐5+2026 (database version 5.2602, software version 4.26.0.6) released by ICDD (International Centre for Diffraction Data, USA) [9]. Rietveld refinements were carried out using the GSAS‐II package version 5365 [10], refining the Chebyschev polynomial background profile, lattice constants, atomic coordinates, scale factors and crystal size parameters. The Crystallographic Information Files (cif) files for the Rietveld refinement were generated by PDF‐5+2026. Raman spectroscopy provided a structural complementary with measurements performed on the powdered samples with a Renishaw inVia Raman spectrometer (Wotton‐under‐Edge, Gloucestershire, UK), equipped with an optical microscope and a CCD detector. A He–Ne laser (632.8 nm) was used as excitation source, while the laser power was kept at 15% of its maximum (~300 mW) to prevent possible overheating effects. Finally, the optical properties were evaluated by UV–Vis diffuse reflectance spectroscopy (DRS). Spectra were collected between 200 and 800 nm using a Shimadzu UV‐2401 PC spectrophotometer, employing BaSO_4_ as reference. For the analysis, the photocatalyst powders were diluted with BaSO_4_ at a mass ratio of 0.050:1. The X‐ray photoelectron spectroscopy (XPS) analyses were performed with a VG Microtech ESCA 3000 Multilab, equipped with a dual Mg/Al anode. Un‐monochromatized Al Kα radiation (1486.6 eV) was used as excitation source. The pressure in the analysis chamber was in the range of 10^−8^ Torr during data collection. The constant charging of the samples was removed by referencing all the energies to the C 1s binding energy set at 285.1 eV. Analyses of the peaks were performed with the CasaXPS software.

### 2.2 Photocatalytic Experiments

In order to determine the photocatalytic activity of the prepared materials, three different model reactions were studied. The first model reaction was the photoreforming of an organic species in aqueous solution to obtain H_2_ in the absence of oxygen. In this system the reduction of water occurred forming H_2_, along with the oxidation of the organic sacrificial agent, that is, in our case ethanol. The reaction occurred in aqueous suspension of the semiconductor in a N_2_ saturated atmosphere. The photocatalytic activity was evaluated through photoreforming tests carried out under UV irradiation. The experiments were performed in a cylindrical Pyrex photoreactor (50 mL total volume) containing 35 mL of an aqueous suspension of the photocatalyst. The catalyst loading was fixed at 0.32 g L^−1^, and ethanol (0.39 M) was added as a sacrificial agent. Irradiation was supplied externally from both flat sides of the reactor using two UV LED IRIS 40 lamps, with a maximum emission centered at 365 nm. Under these conditions, the light intensity in the 315–400 nm range, measured with a UVX digital radiometer, was 1450 W m^−2^. Before starting the reaction, the suspension was purged with nitrogen (N_2_) for 30 min in order to remove dissolved oxygen and ensure an inert atmosphere within the reactor. After this preliminary step, the system was sealed and irradiation was initiated. The experiments were conducted both in the absence and in the presence of platinum, used as a cocatalyst at 1 wt% with respect to the photocatalyst. When required, Pt was introduced by adding 112 μL of an aqueous PtCl_4_ solution (1 g L^−1^ in Pt) prior to the degassing step. All measurements were performed at room temperature. The evolution of hydrogen was monitored by gas chromatography using an HP 6890 Series instrument, equipped with a 60/80 Carboxen‐1000 packed column and a thermal conductivity detector. To evaluate the stability of the catalysts, recycling tests were performed. The study was carried out on the TiO_2_ CW‐Ce 3% photocatalyst that, as will be seen later, proved to be the most active. This material was subjected to three consecutive cycles of photoreforming using aqueous ethanol solutions. In particular, at the end of the first two catalytic runs, the solid catalyst was recovered from the reaction mixture by decantation. After removing the supernatant, the catalyst was washed twice with water and subsequently reused in a new photoreforming test. Consequently, by using this procedure the same catalyst underwent three successive catalytic cycles.

The second and third types of reactions model were aimed to oxidize an organic molecule, either in gas–solid regime (2‐propanol) and in liquid–solid one (5‑hydroxymethylfurfural (5‐HMF)). The oxidation of the gaseous 2‐propanol, was carried in a cylindrical Pyrex batch photoreactor of 125 mL. The powder of photocatalyst (100 mg) was distributed at the bottom of the reactor and the closed photoreactor atmosphere was saturated with O_2_ for 30 min. After that, 2 μL of liquid 2‐propanol (ca. 0.2 mM) were injected into the photoreactor. Details on the set‐up are described elsewhere [11]. The photoreactor was irradiated from the top using the UV LED IRIS above described for the H_2_ generation. The photon flow reaching the reactor was 1450 W m^−2^. The irradiated area of the photoreactor was approximately 1380 mm^2^. During the reaction, samples of reacting fluid (250 μL) were periodically withdrawn using a gas‐tight syringe in order to monitor the process over time. The concentrations of the substrate and the corresponding reaction intermediates were determined by gas chromatography, employing a Shimadzu GC 2010 system equipped with a Phenomenex Zebron Wax‐Plus column and a flame ionization detector. Carbon dioxide formation was analyzed using the same instrument setup adopted for hydrogen detection. For the photocatalytic oxidation of 5‐HMF in a liquid–solid system, experiments were carried out in a 150 mL cylindrical batch reactor. The system was externally irradiated by six UV neon lamps (8 W each). The initial concentration of 5‐HMF was fixed at 0.5 mM, under natural pH conditions, while 50 mg of photocatalyst were used for each run. Under these conditions, the incident radiation in the 315–400 nm range was ca. 3.4 W m^−2^. At selected time intervals, aliquots of the reaction mixture were collected, filtered through 0.25 μm membranes to remove the suspended solid, and subsequently analyzed. Quantitative analysis of 5‐HMF and its oxidation products, including 2,5‐furandicarboxaldehyde (FDC), was performed by HPLC using a Thermo Scientific Dionex UltiMate 3000 system equipped with a diode array detector. Separation was achieved with a REZEK ROA Organic Acid H^+^ column, using an aqueous 2.5 mM H_2_SO_4_ solution as the mobile phase at a flow rate of 0.6 mL min^−1^. Calibration curves were established using analytical‐grade standards of 5‐HMF and 2‐propanol (purity > 99%, Sigma–Aldrich–Merck), which were also employed in the photocatalytic tests. All solutions were prepared with ultrapure HPLC‐grade water (resistivity 18.2 MΩ cm) obtained from a Direct‐Q UV purification system (Millipore).

## 3 Results and Discussion

### 3.1 Characterization of the Photocatalysts

**FIGURE 2:**
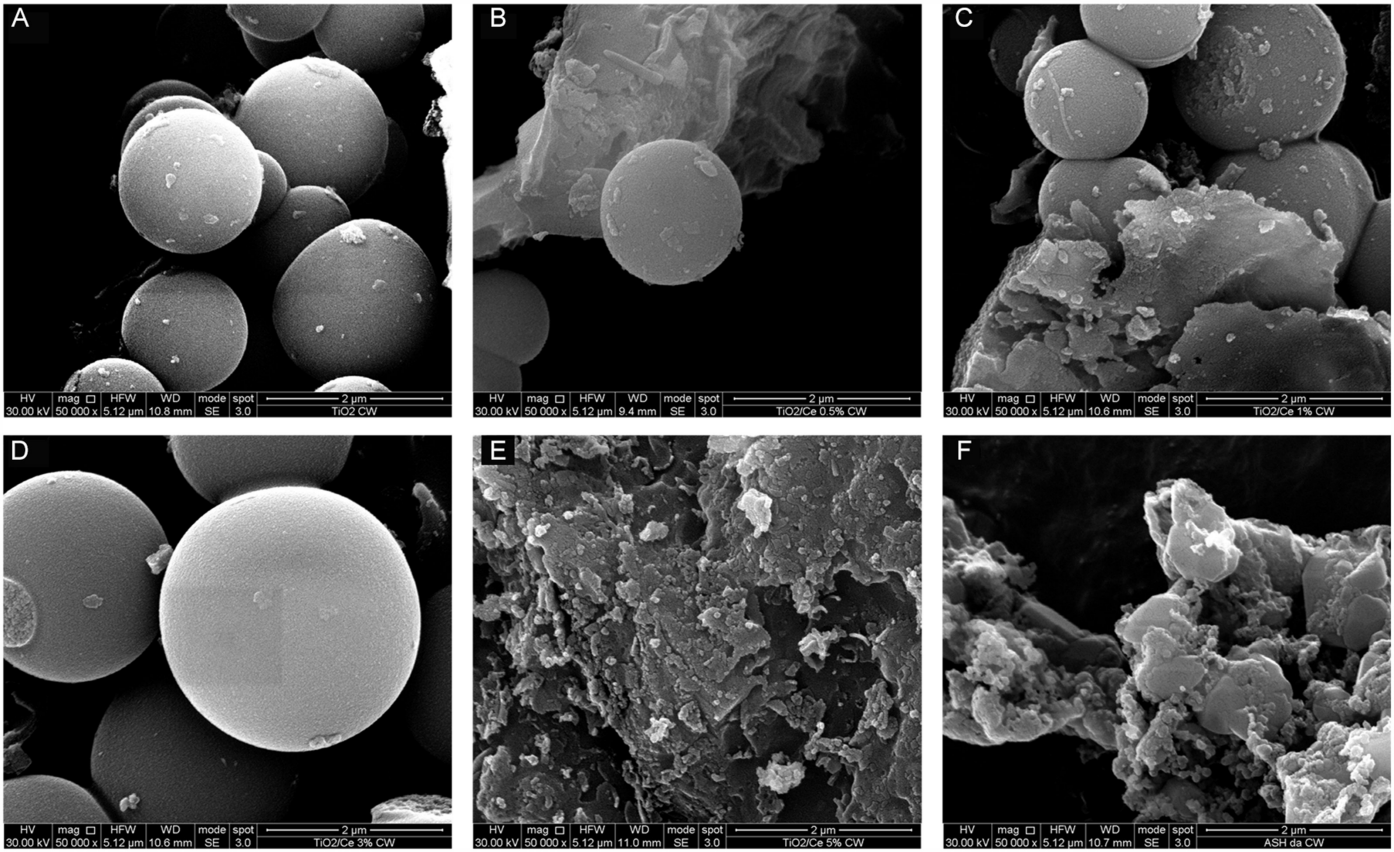
SEM image of the bare photocatalyst TiO_2_ CW (A); TiO_2_ CW‐Ce 0.5% (B); TiO_2_ CW‐Ce 1% (C); TiO_2_ CW‐Ce 3% (D); TiO_2_ CW‐Ce 5% (E); ashes obtained from the calcination of the CW (F).

Figure 2A–F show SEM photographs of the TiO_2_‐based samples along with a picture of the ashes obtained after heat treatment of the CW alone (ASH CW). From the study of the photographs, it is possible to observe that pure TiO_2_ is a nanostructured material with particle sizes ranging from ca. 30 to approximately 40 nm that assemble to form spherical agglomerates with dimensions ranging from ~0.8 to ~1.6 µm. The samples prepared in the presence of the cerium salt show, in addition to these spherical agglomerates, other agglomerates of nanoparticles, also approximately 30–40 nm in size, but with not defined shapes. Furthermore, in the samples with a Ce content of 0.5% and 1%, the spheres have sizes of approximately 1.6 µm, while in the case of the sample with 3% Ce the sizes are larger, up to 2.9 µm. However, in the case of the sample TiO_2_ CW‐Ce 5%, the spherical agglomerates are no longer present. In all cases, by studying the morphology of the samples at higher enlargements (not showed for the sake of brevity) it is possible to observe spaces between the various particles that make up the agglomerates of approximately 6–7 nm, indicative of a certain mesoporosity and in agreement with the results obtained by study of the N_2_ adsorption/desorption isotherms. Finally, the ashes obtained from the calcination of the CW alone are also agglomerates of nanoparticles of approximately 45 nm but with completely random shapes. The SEM photographs described above indicate that the presence of CW in the TiO_2_ precursor not only allows us to obtain nanostructured material but also allows the agglomerates to have a spherical shape. However, when cerium nitrate is also present among the precursors, not all particle agglomerates are spherical. This insight becomes more evident as the Ce content increases. In fact, the TiO_2_ CW‐Ce 5% (sample with the highest amount of Ce) no longer exhibits spherical agglomerates.

The EDX study allowed us to measure the percentage of Ce only in the sample with the highest amount of it (TiO_2_ CW‐Ce 5%). However, the percentage was higher than the nominal one (about 6.5% compared to the nominal 5%) but very close to that obtained through the XPS investigation, see later, which gave a value of 6%. This suggests a slight surface enrichment of CeO_2_ compared to the nominal value. The EDX study of the ash resulting from the heat treatment of CW alone (ASH CW) indicated the presence of metals such as potassium, magnesium, and calcium. These results are in agreement with the XRF analysis, which showed that the inorganic fraction of dried coffee waste is mainly composed of K (21 wt%), Mg (8 wt%), Ca (7 wt%), P (6 wt%), and S (4 wt%), together with traces of Si, Fe, Mn, Cu, Zn, Rb, Ni, Cr, Nb, Sr, Ti, Br, Zr, Sn, Sb, and Eu. The high abundance of alkali and alkaline‐earth elements, particularly K, Mg, and Ca, together with phosphorus and sulfur, is expected to influence the photocatalyst synthesis by affecting crystal growth, particle morphology, surface properties, and the dispersion of the active phase, ultimately contributing to improved photocatalytic performance during photoreforming. These XRF results are consistent with previous reports on spent coffee grounds, identifying K as the predominant inorganic element, followed by Mg, Ca, P, and S, while transition metals occur only in trace amounts [12].

However, the presence of these metals was not detected in the sample containing TiO_2_ nor in the mixtures with Ce probably because the amount of CW ash in these samples is extremely low. Indeed, the ash contained in the final photocatalyst accounts for only about 1% of the starting CW mass.

**FIGURE 3:**
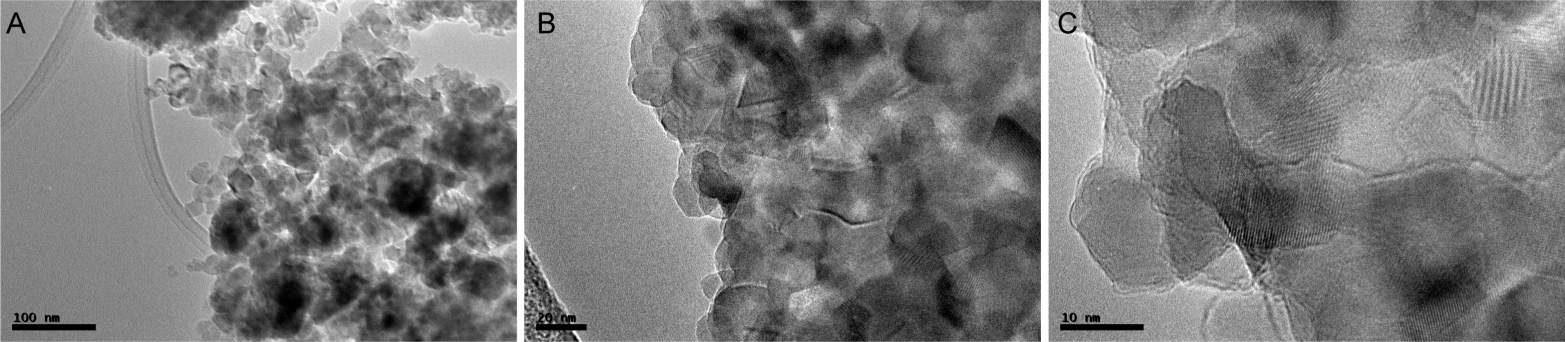
TEM and HRTEM images of the TiO_2_ CW sample acquired at increasing magnifications.

Representative TEM and HRTEM images of the TiO_2_ CW sample are shown in Figure 3. The lower‐magnification TEM image (Figure 3A) reveals that the material consists of agglomerates formed by nanometer‐sized particles, supporting the morphology previously inferred from SEM observations. At higher magnification (Figure 3B), the nanostructured character of the agglomerates becomes more evident, although considerable particle overlap is observed. The HRTEM image (Figure 3C) shows several crystalline domains with different orientations, confirming the polycrystalline nature of the aggregates. These observations corroborate that the micrometre‐sized structures detected by SEM are agglomerates of nanoscale crystalline units.

**FIGURE 4:**
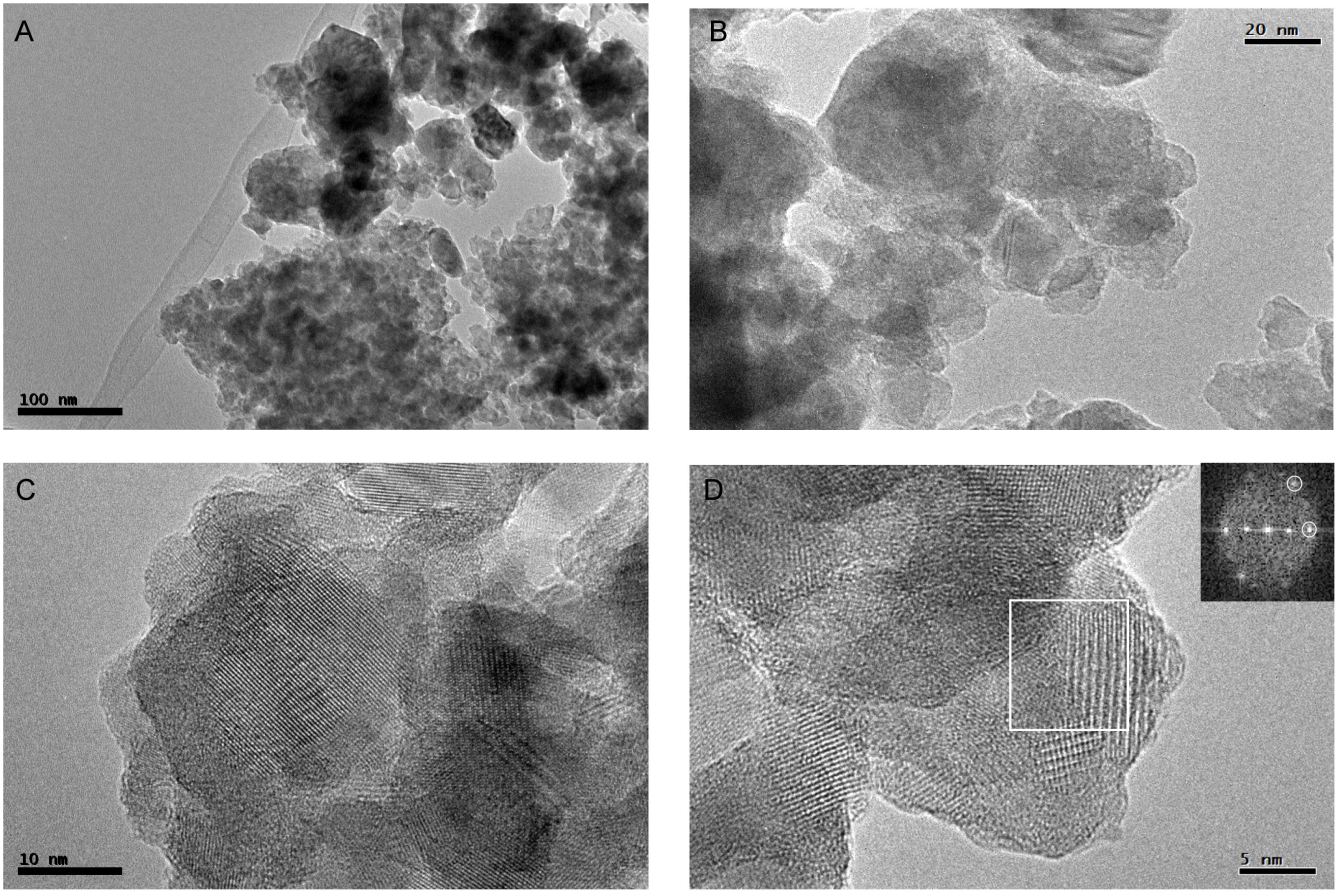
TEM and HRTEM images of the TiO_2_ CW‐Ce 5% sample acquired at increasing magnifications.

The lattice fringes most frequently identified in the examined TiO_2_ CW regions exhibited an interplanar spacing of approximately 0.35 nm, consistent with the (101) planes of anatase TiO_2_. Thus, anatase was the preferentially observed TiO_2_ polymorph at the local scale. Representative TEM and HRTEM images of the TiO_2_ CW‐Ce 5% sample are shown in Figure 4. The lower‐magnification TEM image (Figure 4A) reveals strongly agglomerated nanometer‐sized particles, generally exhibiting irregular or rounded morphologies and lateral dimensions of several tens of nanometers. These observations corroborate that the larger structures detected by SEM correspond to secondary agglomerates rather than individual particles. Compared with the unloade TiO_2_ CW sample, the Ce‐containing material displays a more heterogeneous morphology, with less clearly defined particle boundaries and pronounced overlap between neighboring nanocrystals (Figure 4B). HRTEM image (Figure 4C) reveals well‐resolved lattice fringes extending across differently oriented crystalline domains, together with local Moiré contrast arising from the superposition of crystalline lattices. In addition to the anatase domains identified in the local FFT, lattice spacings of approximately 0.33 and 0.22 nm were observed in other regions of the TiO_2_ CW‐Ce 5% sample. These values are compatible with the (110) and (111) planes of rutile TiO_2_, respectively. The HRTEM results therefore suggest a more heterogeneous crystalline structure for the Ce‐containing material, involving the local coexistence of anatase and rutile TiO_2_ domains together with a periodicity attributable to CeO_2_. This contrasts with the preferential observation of anatase domains in both the TiO_2_ CW and commercial TiO_2_ samples.

**FIGURE 5:**
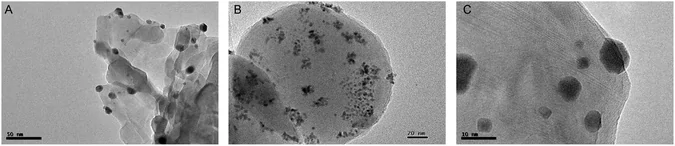
TEM images of the TiO_2_ CW sample recovered after photoreforming reaction with 1% of Pt acquired at increasing magnifications.

The inset of Figure 4D shows the local FFT calculated from the square‐marked region. The circled reflections at approximately 3.19 and 4.20 nm^−1^ correspond to interplanar spacings of 0.313 and 0.238 nm, respectively. These values are consistent with the (111) planes of CeO_2_ and the (004) planes of anatase TiO_2_, supporting their local coexistence within the analyzed region. TEM images of the TiO_2_ CW sample recovered after photoreforming reveal numerous high‐contrast nanoparticles distributed over the TiO_2_ surface (Figure 5A–C). These features were not observed in the pristine TiO_2_ CW sample and are consistent with the Pt deposited in situ during the photocatalytic reaction.

The deposited particles exhibit a heterogeneous size distribution, ranging from a few nanometers to larger particles and small aggregates, and are frequently located at the particle surfaces and edges. Despite Pt deposition, the TiO_2_ support retains its characteristic aggregated and nanocrystalline morphology, with no evident large‐scale restructuring after the reaction. This fact suggests good stability of the catalyst during photocatalytic runs. Moreover, the catalyst stability was further corroborated by reuse tests, which will be discussed later. At higher magnification, lattice fringes remain visible within the support, indicating preservation of its local crystalline order. The deposited Pt nanoparticles may provide electron‐trapping and hydrogen‐evolution sites, consistent with their role as a cocatalyst during photoreforming.

**FIGURE 6:**
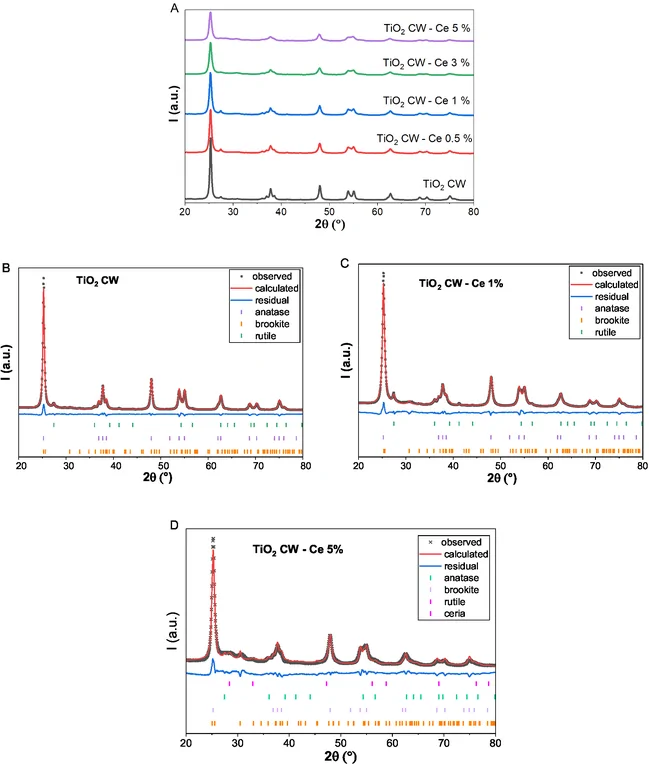
(A) XRD patterns of the five prepared samples and Rietveld refinement of (B) TiO_2_ CW, (C) TiO_2_ CW‐Ce 1%, and (D) TiO_2_ CW‐Ce 5% with the phase 2*θ* positions of ICCD PDF cards.

XRD patterns of the five powders are depicted in Figure 6A, whereas Figure 6B–D show the Rietveld refinements for TiO_2_ CW, TiO_2_ CW‐Ce 1%, and TiO_2_ CW‐Ce 5% as representative examples. The corresponding Rietveld analysis results are listed in Table 1. Rietveld refinement of the XRD patterns revealed that all samples consisted of a polymorphic TiO_2_ system, with anatase (tetragonal, space group I41/amd, ICDD PDF Card—00‐064‐0863) as the predominant phase, in agreement with the HRTEM observation. Variable amounts of TiO_2_ brookite (orthorhombic, space group Pbca, ICDD PDF Card‐04‐007‐0758) and TiO_2_ rutile (tetragonal, space group P42/mnm, ICDD PDF Card—01‐070‐7347) are also present; relative phase fractions are reported in Table 1. Despite the progressive increase in cerium content from 0.5% to 5%, only TiO_2_ CW‐Ce 5% sample exhibits peaks attributable to CeO_2_ (cubic, space group Fm‐3m, ICDD PDF Card—00‐004‐0593). This result is consistent with SEM–EDX results, which detect cerium signals exclusively in the sample containing 5% Ce. The lack of detectable signals in the other samples may be attributed to the low Ce/ceria content associated with small crystallite sizes, which likely renders the phase undetectable by laboratory XRD. Overall, as the cerium content increases, the anatase fraction gradually decreases in favor of brookite [14]. The progressive introduction of cerium also appears to affect the crystallite size, which remains in the nanometric range for all samples, but tends to decrease for the anatase phase and increase for brookite. No clear trend is observed for rutile.

**TABLE 1:** Rietveld refinement results for the investigated samples.

	Anatase	Brookite	Rutile	**CeO**_**2**_	**Reliability** **factors, %**
TiO_2_ CW	Wt% = 82.6 ± 0.9 *V* = 136.33 ± 0.02 Crystal size = 28.7 ± 0.2	Wt% = 10.8 ± 0.9 *V* = 257 ± 2 Crystal size = 3.1 ± 0.4	Wt% = 6.6 ± 0.3 *V* = 62.34 ± 0.06 Crystal size = 17 ± 1	Not present	wR = 5.75 GOF = 3.49
TiO_2_ CW‐Ce 0.5%	Wt% = 78 ± 1 *V* = 136.42 ± 0.04 Crystal size = 20.5 ± 0.2	Wt% = 12.8 ± 0.6 *V* = 259.5 ± 0.3 Crystal size = 17.2 ± 0.1	Wt% = 9.2 ± 0.3 *V* = 62.29 ± 0.06 Crystal size = 17.6 ± 0.9	Not detected	wR = 6.69 GOF = 4.11
TiO_2_ CW‐Ce 1%	Wt% = 71.7 ± 0.8 *V* = 136.33 ± 0.04 Crystal size = 19.9 ± 0.2	Wt% = 18.2 ± 0.7 *V* = 258.9 ± 0.3 Crystal size = 14.1 ± 0.8	Wt% = 10.1 ± 0.3 *V* = 62.19 ± 0.07 Crystal size = 16.1 ± 0.9	Not detected	wR = 6.76 GOF = 4.23
TiO_2_ CW‐Ce 3%	Wt% = 71 ± 1 *V* = 136.67 ± 0.05 Crystal size = 19.1 ± 0.5	Wt% = 22 ± 1 *V* = 260.5 ± 0.5 Crystal size = 14.5 ± 0.1	Wt% = 6 ± 1 *V* = 61.9 ± 0.4 Crystal size = 11.5 ± 0.2	Not detected	wR = 11.88 GOF = 7.47
TiO_2_ CW‐Ce 5%	Wt% = 71 ± 1 *V* = 136.9 ± 0.1 Crystal size = 20.1 ± 0.5	Wt% = 22 ± 1 *V* = 264.3 ± 0.3 Crystal size = 26.1 ± 0.2	Wt% = 3.4 ± 0.8 *V* = 62.3 ± 0.3 Crystal size = 21 ± 3	Wt% = 3.6 ± 0.2 *V* = 160.6 ± 0.8 Crystal size = 7.9 ± 0.8	wR = 10.35 GOF = 6.45

*Note:* Units of measurement: % for phase weight fraction (wt%), Å^3^ for unit cell volume (V) and nm for crystallite size.

Abbreviations: GOF, goodness‐of‐fit [13]; wR, the weighted‐profile R‐factor.

**FIGURE 7:**
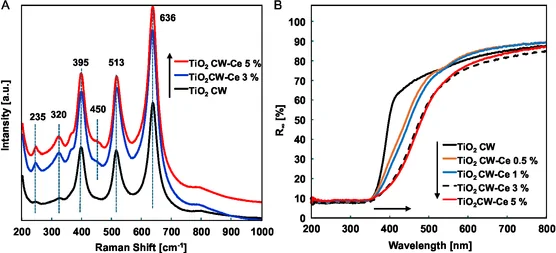
Raman of some of the samples (A) and diffuse reflectance of the materials (B).

Raman spectroscopy of all of the samples shows the five active modes characteristic of TiO_2_ anatase [15], at 144, 197, 395, 513, and 640 cm^−1^, where the last three are shown in Figure 7A. Notably, the presence of an increasing amount of Ce does not modify significantly the spectra of the samples. No transitions relative to crystalline CeO_2_ are observed. The fluorite‐type crystalline ceria presenting a strong transition at 460 cm^−1^ was absent in all of the spectra. Notably, in the presence of Ce (TiO_2_ CW‐Ce 3% and 5% spectra), those peaks appearing at 235, 325, and 450 cm^−1^ can be ascribed to the presence of some TiO_2_ rutile phase. It is worth to mention that FT‐IR spectroscopy confirmed the absence of organic functional groups derived from the biomass waste, as no vibration peak attributable to the templating agent appears, indicating that coffee waste grounds have been completely removed with the calcination. Figure 7B depicts the diffuse reflectance spectra of the photocatalysts. All showed the typical electronic transition representative of the bandgap energy. The redshift of the DRS in Figure 7B observed by enhancing the Ce amount is in good agreement with literature results for similar samples [16]. Tauc‐Plots built by using the DRS spectra allows to estimate the optical band‐gap of the solids (see Table 2) showing a gradual reduction from 3.1 to 2.5 by increasing the Ce content. The catalysts were characterized by N_2_ adsorption–desorption isotherms. According to IUPAC classification, all samples exhibit type IV isotherms with a well‐defined hysteresis loop, consistent with mesoporous materials. The specific surface areas (SSA), average pore diameter and pore size distribution are summarized in Table 2. All catalysts display comparable SSA and narrow, closely matching pore size distributions.

**TABLE 2:** Specific surface area (SSA), textural features and optical bandgap of the materials.

Sample name	Bandgap, eV	SSA, m^2^ g^−1^	Cumulative pore volume, cm^3^ g^−1^	Pore size, nm
TiO_2_ CW	3.1	45	0.10	7.80
TiO_2_ CW‐Ce 0.5%	2.7	45	0.12	7.56
TiO_2_ CW‐Ce 1%	2.7	46	0.12	7.65
TiO_2_ CW‐Ce 3%	2.5	43	0.10	7.70
TiO_2_ CW‐Ce 5%	2.5	42	0.10	7.81

**FIGURE 8:**
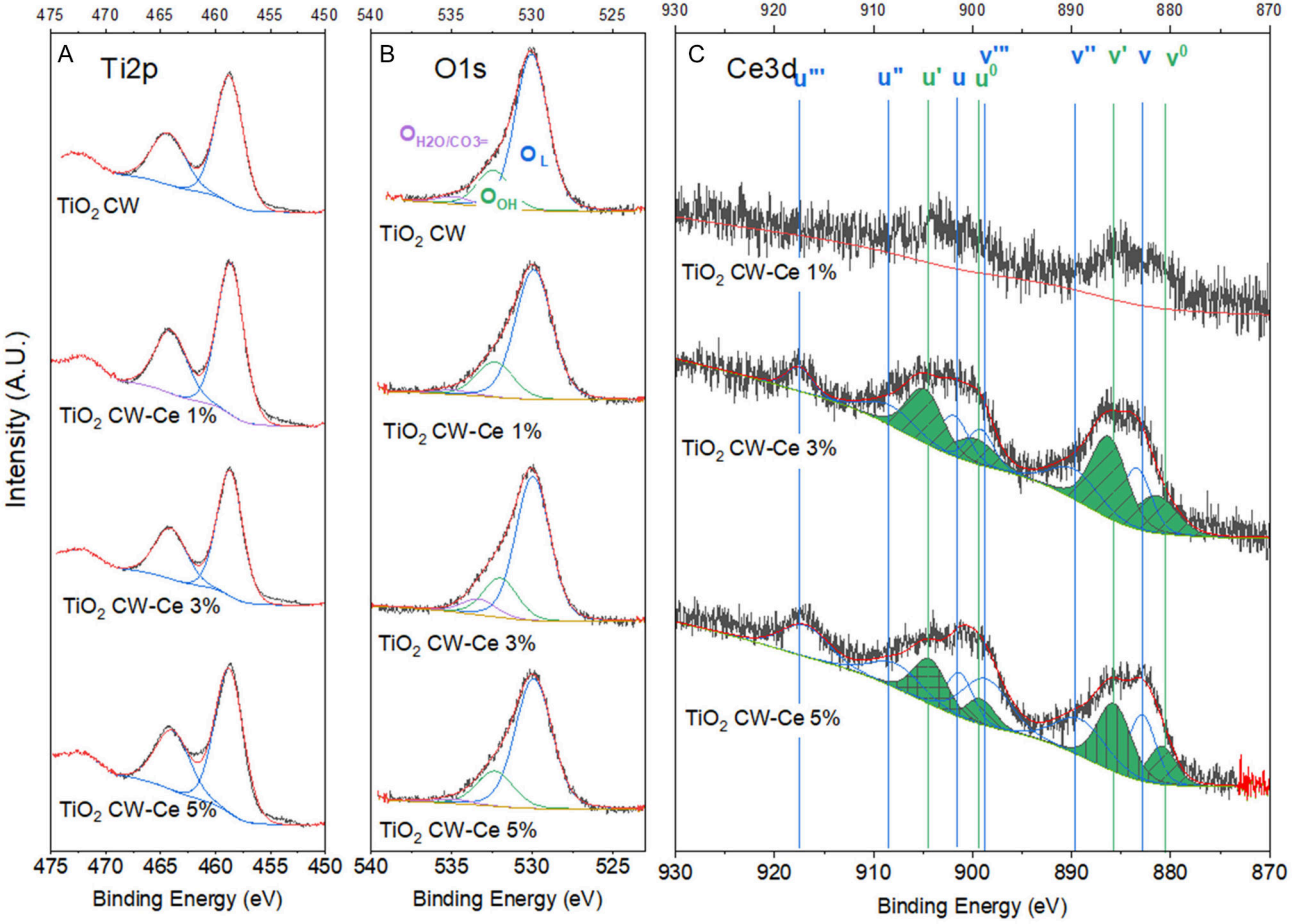
Ti 2p (A), O 1s (B), and Ce 3d (C) XPS region.

XPS analyses were performed on bare TiO_2_ and on samples doped with 1%, 3%, and 5% of cerium. Ti 2p region (Figure 8A) shows a doublet Ti 2p_3__/2_ Ti 2p_1__/2_ separated by 5.6 eV, centered at 458.3 and 463.9 eV respectively, typical of Ti^4^+ in TiO_2_ lattice. At the same time O 1s peak (Figure 8B) has a main component centered at 530.0 eV typical of oxygen in Ti─O–Ti lattice oxygen (O_L_), a component at ca. 532 eV typical of surface ─OH groups (O_OH_) and a minor component at higher binding energy due to H_2_O or carbonaceous (O_H2O/_CO_3_^2^^−^) species chemisorbed on the surface of the materials. No significant shift is observed in the binding energies of the Ti 2p_3__/2_ and O 1s peaks after doping, which can be attributed to the low cerium content [17].

Ce 3d signals for 1%, 3%, and 5% cerium doped titania are shown in Figure 8C along with the deconvolution analysis for the 3% and 5% TiO_2_‐Ce. The signal of the 1% Ce‐doped sample was not deconvoluted due to the low intensity. Ce 3d spectrum is quite complex with the presence of six peaks (denominated *v*, *v*’’, *v*’’’ for Ce 3d_5__/2_ and *u*, *u*’’ and *u*’’’ for Ce 3d_3__/2_) for Ce(IV) and four peaks (*v* ^0^ and *v*’ for Ce 3d_5__/2_ and *u* ^0^ and *u*’ for Ce 3d_3__/2_) for Ce(III) and due to this complexity, quantitative evaluation of Ce(III) and Ce(IV) is tricky [18]. The fitting procedure has been done, taking advantage of the fact that the *u*’’’ peak does not overlap with any other peak and constraining the position of the other 9 peaks to *u*’’’ according to literature [19, 20]. The percentage of Ce(III) and Ce(IV) in the materials has been calculated by the relative amount of *v* ^0^ and *v*’ for Ce(III) and *v*, *v*’ and *v*’’’ for Ce(IV), and the results are shown in Table 3. The presence of Ce(III) increases by decreasing the doping percentage, indicating the formation of cerium titanium oxide compound more than the presence of CeO_2_ domain [21]. This fact confirms the results obtained by XRD analysis, where no CeO_2_ crystalline domains are found up to 5% TiO_2_‐Ce and suggest a stabilization of reduced cerium species associated with Ce–O–Ti interactions and defect formation at the interface. By increasing Ce loading, the formation of larger CeO_2_‐like domains becomes progressively favored [22, 23, 24]. It is worth noting that, although the Ce 3d signal relative to TiO_2_‐1% Ce has not been deconvoluted, the absence of u’’’ peak at ca.917 eV confirm a very high percentage of Ce(III) on this sample.

**TABLE 3:** O 1s binding energy (eV), XPS derived Ce(III), and Ce(IV) relative percentage (%) and Ce/Ti atomic ratio.

Sample	O 1s	Ce(III)–Ce(IV)	Ce/Ti
TiO_2_ CW	530.0 (78%) 532.4 (18%) 534.0 (4%)	—	—
TiO_2_ CW‐Ce 1%	530.0 (80%) 532.2 (17%) 533.5 (3%)	—	0.006
TiO_2_ CW‐Ce 3%	530.0 (73%) 532.0 (19%) 533.3 (8%)	52%–48%	0.02
TiO_2_ CW‐Ce 5%	530.0 (77%) 532.3 (21%) 534.8 (2%)	37%–63%	0.03

The photocatalytic hydrogen production from ethanolic aqueous suspensions of the bare TiO_2_ and composites under UV LED irradiation reveals different results in the absence and in the presence of Pt as cocatalysts. Indeed, the use of a noble metal is a common strategy to enhance the H_2_ productivity in the photo reforming reaction [25, 26]. Table 4 summarizes the H_2_ productivity obtained in photocatalytic reactions performed with the prepared materials, both in the absence and in the presence of 1 wt% Pt. Furthermore, the catalytic activity of these materials was compared with that of a commercial titanium oxide, Huntsman TiO_2_. The catalytic performance of this reference sample is also reported in Table 4. The catalytic performance is expressed in terms of hydrogen evolution rate per unit mass of photocatalyst, along with the apparent quantum efficiency (AQE), calculated according to Equation (1)

**TABLE 4:** H_2_ productivity achieved during the photoreforming of aqueous ethanol solutions under UV irradiation using different photocatalysts, in the absence and presence of Pt, together with the corresponding apparent quantum efficiency (AQE).

Sample	H_2_, µmol h^−1^ g^−1^ no Pt	AQE, % no Pt	H_2_, µmol h^−1^ g^−1^ with Pt	AQE, % with Pt
TiO_2_ CW	47.0	0.01	6288	2.0
TiO_2_ CW‐Ce 0.5%	110	0.03	2286	0.80
TiO_2_ CW‐Ce 1%	181	0.06	1484	0.50
TiO_2_ CW‐Ce 3%	312	0.10	540	0.20
TiO_2_ CW‐Ce 5%	90.0	0.03	288	0.08
TiO_2_ Huntsman	0.00	0.00	8200	2.6

(1) $$\mathrm{AQE}\left(\%\right)=\frac{2\cdot (\text{moles of evolved hydrogen})}{\text{moles of incident photons}}\cdot 100$$

where the moles of hydrogen evolved correspond to the amount formed over the entire reaction time and accumulated in the reactor headspace, and the moles of photons correspond to those emitted by the LEDs (at *λ* = 365 nm) and incident on the reactor surface throughout the duration of the reaction.

In the absence of Pt, increasing the Ce content enhanced H_2_ productivity, with the TiO_2_ CW‐Ce 3% photocatalyst exhibiting the highest activity (312 µmol h^−1^ g^−1^). This behavior can be attributed, in agreement with the literature, to the beneficial effect of cerium doping, which promotes charge separation and surface reactivity through Ce^3+^/Ce^4+^ redox cycling and the formation of oxygen vacancies [16]. However, a further increase in Ce content (5%) led to a decline in activity, likely due to the formation of recombination centers or surface passivation effects. The AQE values were relatively low, indicating that a significant fraction of the incident light from the LED source was not effectively utilized for H_2_ production.

The addition of 1% Pt markedly enhanced H_2_ productively, as observed before [26], particularly for pure TiO_2_ (6288 µmol h^−1^ g^−1^). In contrast, Ce‐containing composites exhibited lower performance compared to pristine TiO_2_, with activity decreasing as the Ce content increased. This trend may be attributed to competitive interactions between Pt and Ce sites or to modifications in surface chemistry. These findings highlight the importance of optimizing dopant concentration and cocatalyst synergy. Notably, both H_2_ productivity and AQE values improved by more than an order of magnitude upon Pt addition for most of the catalysts investigated.

Interestingly, commercial Huntsman TiO_2_ was completely inactive in photoreforming in the absence of Pt as a cocatalyst. Conversely, upon Pt addition, its hydrogen productivity became the highest, even slightly surpassing that of the TiO_2_ CW sample.

**FIGURE 9:**
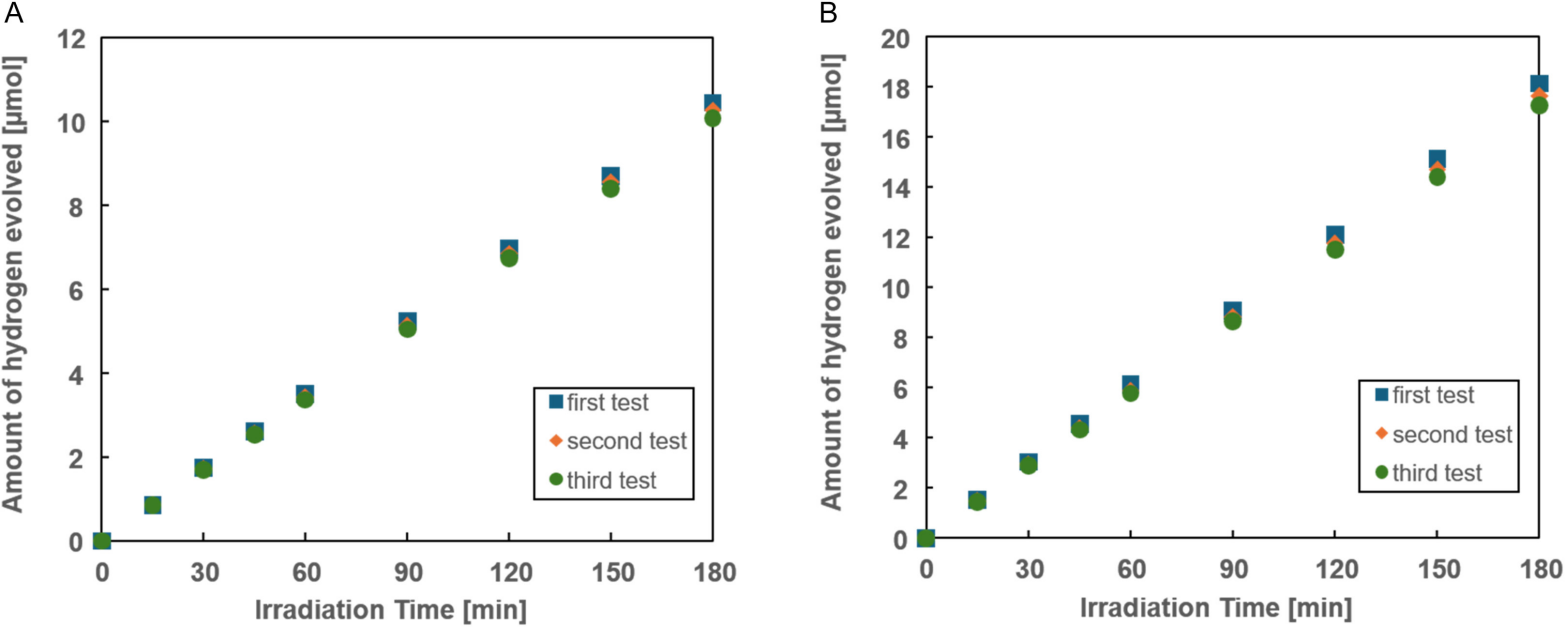
Amount of hydrogen evolved during three consecutive photoreforming test carried out in the presence of TiO_2_ CW‐Ce 3% photocatalyst (A) without Pt and (B) with 1% of Pt as cocatalyst.

To evaluate the catalytic stability, three consecutive reuse tests were performed using the TiO_2_ CW‐Ce 3% catalyst, both in the absence and in the presence of Pt (1%) under photoreforming conditions. The corresponding results are shown in Figure 9A,B.

These experiments indicate that both catalysts retain a substantial level of stability over the three consecutive cycles, exhibiting only a minor decrease in hydrogen production. Specifically, during the third reuse test, the amount of evolved hydrogen decreased by approximately 3% compared to the first run with the fresh catalyst in the absence of Pt and by about 4% when Pt was included as cocatalyst.

**FIGURE 10:**
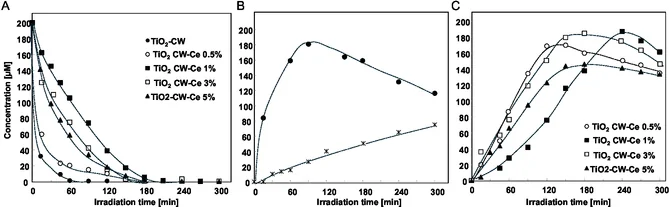
(A) Evolution of the concentration of 2‐propanol during the photocatalytic reaction in the presence of the prepared materials; (B) evolution of acetone (⏺) and CO_2_ (*) in the presence of TiO_2_‐CW; and (C) evolution of acetone in the presence of the composite materials during the photocatalytic experiments. The values reported in the graphs represent the average of three measurements. The deviation of each value from the mean was in all cases below 2%.

The photocatalytic degradation of 2‐propanol in the gas–solid regime was investigated using the prepared solids as photocatalysts, as shown in Figure 10A. Each experiment was performed in triplicate, and the values reported in the graphs correspond to the average of the three measurements. In the presence of TiO_2_ CW, a gradual oxidation of 2‐propanol was observed, accompanied by the formation of acetone as the main oxidation product (Figure 10B) and by a certain amount of CO_2_, indicating partial mineralization of the substrate. The presence of Ce in the photocatalysts initially slowed down both the degradation of 2‐propanol and its conversion into acetone for samples containing 0.5 and 1 wt% Ce (Figure 10A,C). In contrast, increasing the Ce content to 3 and 5 wt% reversed this trend, leading to faster reaction rates. In all cases, no CO_2_ was detected for the Ce‐containing samples, suggesting a lower oxidizing ability of these materials compared to pristine TiO_2_. The TiO_2_ CW‐Ce 1% photocatalyst exhibited the lowest oxidizing activity, displaying the weakest 2‐propanol degradation and the slowest acetone formation. The presence of Ce significantly diminishes the oxidizing capability of the composites, being the Ce 1% the least active. The comparatively higher activity observed for the 3 and 5 wt% Ce samples likely results from the presence of the rutile phase (observed by Raman) enhancing the photocatalytic performance of the composites.

**FIGURE 11:**
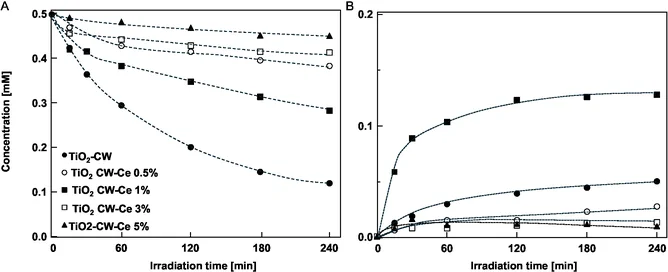
Photocatalytic oxidation of 5‐HMF (A) and formation of FDC (B) in the presence of the prepared materials. The values reported in the graphs represent the average of three measurements. The deviation of each value from the mean was in all cases below 2%.

The oxidative behavior of the materials was further investigated through the oxidation of 5‐HMF in aqueous suspension of the photocatalysts to evaluate their ability to promote the selective partial oxidation toward 2,5‐furandicarboxaldehyde (FDC). Also in these case, each experiment was performed in triplicate, and the values reported in the graphs of Figure 11A,B correspond to the average of the three measurements.

As reported in Figure 11, TiO_2_ CW is the most active catalyst, followed by the TiO_2_ CW‐Ce 1%. Interestingly, the catalyst exhibiting the highest oxidizing activity in 2‐propanol oxidation also proved to be the most oxidant toward 5‐HMF degradation, albeit the highest selectivity to FDC is assigned to the TiO_2_ CW‐Ce 1%, which is less oxidant toward the 5‐HMF substrate. This result suggests that a lower overall oxidizing strength is beneficial for steering the reaction pathway toward aldehyde formation rather than complete oxidation, highlighting a favorable balance between activity and selectivity for this material, as shown in Figure 11. The partial oxidation of 5‐HMF using the composite photocatalysts reveals the intrinsic photocatalytic behavior of the materials. The formation of FDC follows a similar trend for all of the photocatalysts, but TiO_2_ CW‐Ce 1% promotes the highest yield, suggesting lower efficiency of 5‐HMF conversion but higher selectivity toward the partial oxidation product. In contrast, enhancing amount of Ce in the composite diminished the activity of the solid, likely due to excessive Ce content leading to recombination centers or surface passivation. These findings underscore the importance of fine‐tuning cerium content to optimize both conversion efficiency and product selectivity in photocatalytic biomass valorization.

## 4 Conclusion

Ce‐modified TiO_2_ photocatalysts were synthesized through a simple one‐pot method, using spent coffee grounds as a template in the absence of any solvent. The structural characterization revealed that samples were anatase TiO_2_ with well‐dispersed cerium species. The higher cerium loadings promoted rutile formation and induced a redshift light absorption, resulting in a reduced bandgap of the solids. Pure TiO_2_ is made up of ~30–40 nm nanoparticles that organize into fairly uniform spherical agglomerates (~0.8–1.6 μm). When Ce is introduced, this ordered structure gradually changes: the aggregates become larger and less regular, and at higher loadings (5% Ce) the spherical morphology is no longer observed. In all cases, small voids between particles (~6–7 nm) point to a mesoporous structure. The presence of Ce(III) increases by decreasing the Ce percentage indicating the formation of cerium titanium oxide, rather than CeO_2_. Cerium incorporation strongly affected the photocatalytic activity, but the trend depended on the reaction type and regime. Under anaerobic conditions, the composites enabled efficient H_2_ production from aqueous ethanol photoreforming without the need for noble metal Pt‐cocatalyst, demonstrating enhanced reduction capability with respect to that of bare TiO_2_ at least up to the amount of 3% of Ce. Conversely, in the presence of Pt, as a cocatalyst, the most active material was bare TiO_2_. Stability tests were performed on the TiO_2_ CW‐Ce 3% photocatalyst, which proved to be highly stable and readily reusable, as evidenced by the nearly constant hydrogen production observed across the three catalytic cycles investigated.

Under oxidative gas‐solid conditions, the overall oxidizing power decreased when Ce was present in the TiO_2_‐ based composite materials, reaching a minimum oxidant ability for the 1 wt% Ce in the case of 2‐propanol oxidation and for the 5 wt% Ce in the case of 5‐HMF oxidation. This different behavior of the materials can be due to the different type of molecule to be oxidized, the different reactor used, as well as the different intensity of the UV radiation used. However, the reduced oxidizing strength can be advantageous for selective oxidation. In particular, the conversion of 5‐HMF to FDC in aqueous suspensions of the Ce‐containing photocatalysts compared to bare TiO_2_ gave the idea of this oxidation ability. Indeed, the sample containing a 1% of Ce was less oxidant than TiO_2_ albeit was the most selective versus the formation of the partial oxidation product. The results evidenced that controlled cerium incorporation provides a strategy to tune the redox properties of TiO_2_ toward selective oxidation and reduction processes. This waste‐derived, solvent‐free synthesis provides a scalable and sustainable way to produce multifunctional photocatalysts while valorizing biomass and aligning with the principles of green chemistry and the circular economy.

## Conflicts of Interest

The authors declares no conflicts of interest.

## Data Availability

The data that support the findings of this study are available from the corresponding author upon reasonable request.
